# Outstanding women psychologists mainly from Europe – What helped and what limited them in their scientific careers? Guidelines for gender equity programs in academia

**DOI:** 10.3389/fpsyg.2022.877572

**Published:** 2022-09-06

**Authors:** Beata Pastwa-Wojciechowska, Aneta Chybicka

**Affiliations:** Institute of Psychology, University of Gdańsk, Gdańsk, Poland

**Keywords:** women, psychology, scientific career, successes, dilemmas

## Abstract

The manuscript is based on a series of structured interviews with female scientists from around the world who have made significant contributions to psychology and have an impact on their cultural areas. The authors interviewed female scientists and researchers from a similar age group, but from different regions of the world, to capture the factors influencing careers of interlocutors from a similar period and enabling cultural inference. Both the universal and the cultural barriers faced by female scientists/researchers in career development and the factors that have contributed to success in psychology are discussed. Universal and cultural factors served in this manuscript as a guideline for gender equality programs in academia to overcome gender stereotypes, support early career development, support women in reaching leadership positions, and enhance women’s visibility.

## Introduction

The contribution of women in Polish scientific life is prominent, if only because they outnumber men. However, their representation decreases at the later stages of their scientific careers. The same is true about them performing administrative functions in universities, such as directors of institutes, deans, or rectors. Ironically, even though they have more time and space to focus on their professional careers with age, women constitute a small percentage of university management. When analyzing the list of influential figures shaping the history of universities, it can be noticed that the dominant role in this aspect also belongs to men, both in the organization and the development of scientific life. The history of the establishment and development of many Polish higher education institutions shows that men are considered significant figures. They are acknowledged for their specific achievements, regardless of the historical period – at old, renowned universities with a rich history (such as The Jagiellonian University – established in 1364) or much younger ones (e.g., The University of Gdańsk – established in 1970). Therefore, many questions arise, such as: What happens in the course of women’s professional and personal lives that they become less and less visible over time? Is it the specificity of functioning in scientific institutions of just Polish women, or do women from other countries have similar experiences? What prevents women from being visible in “masculine” universities? Do they have the desire and willingness to participate in university management structures?

These questions seem to be significant for the proper understanding of women’s role and contribution to science, whereas the present study focused on describing the career path of women scientists in the field of psychology. Therefore, one may well ask whether the abovementioned questions are universal for the career path of women in science or whether other aspects should be taken into account in the case of women psychologists. Lastly, how to present our interviewees as persons of particular significance for their environment while being representative of the scientific community of psychologists? We discovered further questions that became our scientific and personal inspirations throughout the planning and implementation of the project.

As our starting point of the analysis, we decided to choose women of renowned academic and professional status in psychology. By exploring their history of work at university, we recognized their academic paths and understood ourselves and the environment in which we operate. In other words, we examined the views of women psychologists on their experiences and barriers they had to face to participate in professional life in the field of psychology fully.

### Women in science: Biography and self-narration as the methods of self-exploration and source of social knowledge

Saying that science plays a crucial role in societies’ lives may sound cliché, but both science and social life indeed undergo numerous changes. In the context of analyzed problems, the relevant questions to be asked include: (a) how women’s participation, visibility, and marginalization contributed to the development of knowledge, and (b) how the culture and content of science are based on the contemporary relations between genders (Wyer, 2018). These questions are essential because they became the subject of scientific research (Ceci et al., 2009; Ceci and Williams, 2011; Chybicka and Zubrzycka, 2015) and analyses conducted by various institutions (such as data from European Union Member States). Ceci and Williams (2011) point out that the focus on gender discrimination is a misplaced effort. This is because society is then engaged in solving the past problems rather than addressing the significant limitations that discourage women from participating in today’s scientific careers. As a discipline, psychology deals directly with gender as a theoretical construct and addresses women’s insufficient representation in science (Rutherford, 2020). Women can be the “object” of research (Osbeck, 2020), and gender can be a category that constitutes research (Febbraro, 2020; Rutherford, 2020). That is why this paper aims to investigate how women, being psychologists, perceive their career paths and gender issues throughout their scientific growth.

A significant number of scholars represent the approach defining biography as a social phenomenon (Hałas, 1990; Stemplewska-Żakowicz, 2002; Chase, 2009; Wengraf, 2012), constituting a particular part of social reality, however, captured in its individual categories (Włodarek and Ziółkowski, 1990). Therefore, we must consider human life in terms of the unique phenomenon of psychophysical functioning in a specific period of a lifetime and the sociocultural determinants of the influence of social time (Piorunek, 1996). The reality perceived in a diachronic way offers an individual a wide range of social situations, which can only be handled through appropriate action competencies (Hurrelmann, 1994). According to Hoerning (1991), transformations in the biographical project occur because of social opportunities and the individual’s attributed and acquired capital and life experience. Thus, Hoering uses the term “social opportunities” for socially established patterns and interpretations that may help cope with events and situations happening in life. The individual’s life experience, on the other hand, is seen in terms of biographical memory. Therefore, we understand an individual biography “as a sequential and complementary whole in which biographical phenomena happening at particular stages of life are always interrelated to the past and the future. The course of a person’s life and their development constitute a complex whole in which each of the major biographical periods is linked to the subsequent periods, and the life tasks and biographical experiences of the temporary life stages are relevant to and affect the entire human biography” (Tyszkowa, 1988, p. 14). It should also be remembered that the course of a person’s life is strongly influenced by environmental factors (the activities that regulate relations with the environment must also take into account the requirements and limitations of that environment) and by culture, which promotes specific models of life and consequently provides criteria for the acceptance or the lack of acceptance of the chosen life path. From our point of view, another important term was the concept of experience, which is defined as a change modifying factor in human psychology, behavior, or personality. Therefore, to outline this context, the term of a cultural and personal set of stories (as a kind of cultural background and heritage) and situational determiners (significant social circumstances) is introduced. These notions constitute the so-called psychological space (Hänninen, 2004), in which the discourse moves from the collective and public sphere to the individual and private one. Every culture has some stories that are more powerful than others – they are said to be privileged, such as dominant or master narratives. Other stories are silenced and marginalized (such as counter-narratives) because otherwise, they would reveal and promote alternative interpretations of reality. A personal set of stories, on the other hand, constitutes the narratives preserved in one’s memory about the person’s experiences, as well as the narratives adopted from a cultural heritage that became “personalized.” When analyzing biography, one should also consider situational factors that constitute the current life circumstances and the various opportunities, resources, and constraints – circumstances partly created by them and partly being beyond their control.

The model of narrative circulation – a story about one’s own experience told, for example, to junior academic staff members enriches the cultural collection of stories because it can be transmitted to the next generations and contribute to the experienced narrative of life.

## Materials and methods

### Procedure

In the first place, criteria were established for selecting university-based female psychological scientists, authority figures in their professional environment, namely the development of psychology, in their countries. We based our list on our understanding of the psychological environment and discussions with people from different centers and countries. We then asked the designated individuals for their consent to conduct the interview, stating its purpose and mode as well as how the data would be collected, processed and used. As a result, ten women psychological scientists agreed to participate in the interviews.

Due to the distance between interviewers and interviewees as well as the COVID-19 pandemic, the interviews were conducted as recorded meetings on the Zoom or Teams platforms. The interviews were conducted between October 2021 and December 2021.

The interviews lasted approximately 2 h, and all were recorded in audio format with the laptop’s camcorder. The study participants were informed of this fact beforehand and all consented. They were also informed of the purpose and scope of the study.

The study authors prepared transcripts of interviews (and the recordings were subsequently deleted).

To address ethics considerations, when recording the interviews, the authors asked each study participant whether they agreed to include parts of their statements in the final text and whether they agreed to include their personal data, such as their name, affiliation and country of origin. This has also been confirmed in writing by each participant. They all consented to disclose their names and affiliations, and they also acknowledged the form in which this information was to appear in the text. The authors believe that the fact that all women scientists agreed to include their names lends credibility to the study and allows the reader to assess more objectively the data collected and the conclusions presented. Readers can also find more information about those exceptional women scientists interviewed and their impact on their communities.

To ensure rigor in the presented qualitative study, the authors followed the standards for the quality of the research process (Ćwiklicki and Urbaniak, 2018). They included:

- Transparency – the authors ensured that the study process and procedure were clearly described to the participants.

- Reliability (repeatability of the study procedure) – to ensure the study reliability, each participant was asked a set of 19 questions in five categories (motivation to work as a psychological scientist, motivation to combine theory with practice, the most important scientific and practical achievement, what helped study participants in the development of their scientific career, barriers for women in the field of psychology, and guidelines and recommended actions to promote equal opportunities for women in science). Those interested in repeating the study can obtain the interview questions from the authors to ensure complete repeatability of the study. In particular, it would be interesting to see if other researchers wished to conduct this study for non-psychology sciences or other population of women scientists.

- Reflexivity – the authors know that their presence during the study can influence the results. This risk was reduced by the authors who remained out of the study participants’ sight during the interviews. The authors, as the right moments, asked further or in-depth questions to obtain comparable data from all study participants.

- Credibility – the authors ensured that study participants came from different countries and backgrounds but showed research-relevant characteristics, such as being women, dealing with the practical and scientific aspects of psychology, being employed in the academic field and having remarkably positive impact on their communities. This aspect is discussed in more detail in the Participants section. Due to more accessible Polish women psychologists, the over-representation of subjects is of Polish origin. However, they are persons with a great deal of experience in international work. Of course, such a selection of participants imposes limitations on the interpretation of the results obtained, particularly in terms of cultural factors. This aspect is further analyzed in the text and discussed in the Results section.

### Participants

The study group consisted of ten women selected based on the following criteria: (1) gender - female, (2) psychological education, (3) profession – academic teacher, (4) combining scientific and practical work, (5) outstanding achievements in the field of practical psychology and science (Table 1).

**TABLE 1:** The list of persons who participated in the study, including their affiliations, academic titles, country of origin and their expertise area of psychology.

Prof. Gopa Bhardwaj	Delhi University, ex. Dean	Delhi	India	Social and cultural psychology
Prof. Maria Beisert	Adam Mickiewicz University	Poznań	Poland	Clinical psychology and health psychology
Prof. Eleonora Bielawska-Batorowicz	University of Łódź	Łódź	Poland	Clinical psychology and sexology
Prof. Antonia Bifulco	Middlesex University	London	United Kingdom	Lifespan psychology
Prof. Marta Bogdanowicz	University of Gdańsk	Gdańsk	Poland	Developmental psychology
Prof. Lidia Cierpiałkowska	Adam Mickiewicz University	Poznań	Poland	Clinical psychology and health psychology
Prof. Márta Fülöp	Research Centre of Natural Sciences, Karoli Gaspar University of the Reformed Church	Budapest	Hungary	Social and cultural psychology
Prof. Bernadetta Izydorczyk	Jagiellonian University	Cracow	Poland	Clinical psychology and crisis intervention
Tina Lindhard, Ph.D.	International University of Professional Studies, President CCA Spain, Chair of Consciousness Research, CICA International, Council of European Grandmothers	Spain	Madrid	Consciousness research
Prof. Stanisława Steuden	John Paul II Catholic University	Lublin	Poland	Personality psychology and clinical psychology

The study group consisted of women from different countries: Poland, Hungary, Spain, Great Britain, and India. In addition, we chose participants with different outlooks, religions, and cultural backgrounds. The study aimed to diagnose the universal experiences of women in the field of psychology.

Due to easier access to Polish women scientists in psychology, half of the participants are of Polish origin. However, they are persons of extensive experience in international work. Of course, such a selection of participants imposes limitations on the interpretation of the results obtained, particularly in terms of cultural factors, which will be further analyzed in the text.

### Instruments

The data was collected using a psychological, structured interview. This tool is characterized by several features: (1) given the scope of the content discussed, this was an indepth interview which referred to the personal experience of the study participants, their autobiographical experiences observed from both an individual and a subjective perspective (Stemplewska-Zakowicz and Krejtz, 2005), and (2) because of how it was structured, the interview should be considered as an open interview, built on contact, but with a hidden structure, determined by the narrative theme (Stemplewska-Zakowicz, 2010). The interview scenario consisted of 19 questions, supplemented, if necessary, by supporting or clarification questions asked depending on the content of the statements. The questions referred to five areas: (1) motivation to work as a psychological scientist, motivation to combine theory with practice, (2) the most important scientific and practical achievement, (3) what helped study participants in the development of their scientific careers, (4) barriers for women in the field of psychology, and (5) guidelines and recommended actions to promote equal opportunities for women in science.

### Results

After transcribing the interviews, extracts relating to the issues under consideration were selected.

Next, the statements were allocated to different categories based on their content, which allowed establishing the structure of the analysis. Finally, the most representative statements were chosen and included in the analysis below. The quoted statements capture as many diverse opinions as possible and, importantly, the most common threads in the interviews. We deliberately left the informal language of the study participants in the publication, reflecting their uniqueness and the intimacy of communication during the interviews. Unfortunately, due to the limited length of the article, we were not able to quote the statements of all study participants in all five categories of qualitative analysis.

The results are presented in five parts, according to categories of questions used during the interviews and resulting from the obtained data.

1. Motivation to work as a psychological scientist, motivation to combine theory with practice,
2. The most important scientific and practical achievement,
3. What helped study participants in the development of their scientific career,
4. Barriers for women in the field of psychology, and
5. Guidelines and recommended actions to promote equal opportunities for women in science.

## Results

The results are presented in five categories, resulting from the assumptions for the research rigor discussed above. To ensure the study’s reliability, participants were asked 19 questions relating to those five categories. For clarity, the results are also presented in five categories.

### Motivation to work as a psychological scientist, motivation to combine theory with practice

In the first area of analysis, the most frequently repeated statements of the study participants referred to their non-egocentric motivation stemming from values such as universalism and benevolence. They expressed the desire to bring valuable and helpful things to the lives of others, to share something that they discovered and considered to be important, courageously promote unpopular knowledge, despite the risk of being intimidated in the scientific world, and a desire to care for their families. Out of the ten participants, none said their motivation was to purely contribute to science or earn the prestige associated with work at a university. Participants were not guided by a self-centered motivation to engage in learning, be promoted, or win something for themselves. Instead, they saw their work as a form of caring for others or a mission.

Prof. Márta Fülöp

I did not want to be a scientist. I did not want to be a scientist at all. I wanted to be a practicing psychologist, a clinical psychologist. I wanted to treat people and understand the disease. I started a researcher job as a student for money. Later, I got pregnant with my first child, and I could not work 8 h a day, 5 days a week in a hospital. Neither did I want to send my child to the nursery. When he was 1 year old, I got a phone call from the Hungarian Academy of Sciences that they were looking for a young scientist and wanted me to apply. That is how I become a researcher and a scientist.

Tina Lindhard, Ph.D.

My field is human consciousness, and my interests include death, drugs, dreams, and meditation. Having deep spiritual experiences and being trained to explore the nature of consciousness, I want to bring this knowledge into science. So my motivation is to maybe introduce a new aspect to science, to bring in the idea of a mystical scientist. Moreover, I am not alone; there are other women working in this field. Because we realize that by choosing our outer senses, we cannot open the universe’s secrets. You can only begin to get this higher inspiration by going inside, and Einstein was well aware of this.

I feel we all look at this mystery we are a part of through different lenses – and in the end, all we can do is lend somebody the lenses through which we are looking at reality, and they can do the same with us.

However, in my opinion, only when we combine the “inner and outer science,” will we make many breakthroughs. Einstein said, “the world that we have made as a result of the level of thinking we have done thus far creates problems that we cannot solve at the same level as the level we created them” (Einstein as cited in ICARUSFALLING, 2009, para. 1). We created these problems by using our thinking mind and consciousness, so using that will not solve our problems. Therefore, my motivation and the next step forward involves the mystical inner journey where we connect with the deeper intelligence in us *via* our hearts, which, when the searcher is sincere, reveal inspirational new insights that can never come through our intellectual mind.

Prof. Maria Beisert

My motivation for research and practical work is to deal with things that involve a specific research risk, the risk of non-acceptance, things that introduce some ferment. I am not afraid of that. Because there is nothing to lose, there will be discussion and noise around the issue, which can only be used to introduce the topic into the space. What I am interested in is their withdrawal. Their withdrawal, displacement, or denial causes specific pathological arrangements to be developing and destroy human lives. My motivation is not to deal with something unusual or pathological, and I do not think that is great. I believe that this requires discovery, just as a mine requires disassembly, and then it is forbidden to build something completely different from the individual parts of the mine.

Prof. Lidia Cierpiałkowska

At the beginning of my professional career, my motivation as a scientist was how psychology might explain the everyday functioning of a human being. When the time came for my professional practice, I chose to have my field practice during my fifth year of studies at a detox clinic. However, on the first day there, I felt horrified because, in Poznań at those times (the breakthrough of 1972/73), the place was extremely gloomy. I listened to various conversations in the psychologist’s office during my practices. It was then that I asked myself for the first time: “Why are some people getting treatment and others dying of addiction?”

It was only later that I thought about explaining why some people behave as they do in a given situation while others behave differently. In addition, it must be understood that these were times in psychology when, if one asked, “Why does a person react in this or another way?,” the answer was that the brain was structurally in good or bad form. In Poland at that time, biological arguments were used to explain things, and the psychology of personality was hardly acknowledged. Perhaps there were few scientific publications on personality psychology available then to us. Thus, the question of why a person behaves in a given manner was challenging in the light of the knowledge of those times.

The analysis of the study participants’ statements indicates that their internal motivation, although varied, involved reaching personal goals, which allowed them to practice values such as self-fulfilment, competence development, independence, and accountability. Their pursuit of professional activities was affected by factors such as the need to address specific problems, including exploring the responses to behavioral mechanisms. Some of the study participants referred to their personal situations (e.g., reconciling the role of mother and scientist) or opportunities for developing scientific interests (e.g., limited or marginal access to literature).

### Key achievements

When replying to the question on crucial achievements, none of the study participants mentioned the administrative position that most held. Instead, they all drew attention to the practical translation of their scientific activities. They also mentioned the importance of their scientific discoveries for human functioning and understanding of behavior in the light of their research results. It was also interesting that half of the study participants pointed out that they had been asked to carry out administrative functions (dean, head of the department, organizing a new academic and research unit) because the situation was challenging and problematic, and someone was needed to deal with it. It was the same when things had to be organized from scratch or a new entity was to be established. Women in science often seem to be valued for their hard work in very demanding conditions. The statements of the study participants that illustrate the issue described are presented below.

Prof. Antonia Bifulco

I was trying to take science to practice. The really good link was the methods. I have developed an interview of child abuse and was using that to inform social workers on conditions of child abuse, and they benefited a lot from that. I also worked much time with the attachment model. The issue is that if you had an adverse childhood, you are more likely to have distorted attachment patterns, which causes lots of problems in making relationships later. You can classify these attachment problems into two different styles, and I developed an interview for doing that. It gave a lot of people rationale for where they were, which is easier to work with. Again social workers and clinical psychologists find it a useful way of assessing attachment. In the exchange of knowledge between scientists and practitioners, both sides benefited a lot. To train social workers to use an interview where they have to score according to prescribed categories, they find it useful to help them decide how to categorize somebody. From there, they can decide on care and plan what they should do. We found that you can train most professionals with the right training package; they do not have to be researchers.

Prof. Maria Beisert

I consider dealing with controversial issues my most outstanding achievement in my scientific and practical work. I would mention here four groups of issues: the sexuality of young children, incest, pedophilia, and child masturbation - this is what my next book is about. These topics describe the dark or the undesirable side of human function. In my opinion, it is worth either bringing them out of the shadow, discussing them openly, or letting them contribute to a change. I also think of non-standard thinking that goes across stereotypes. This way of thinking is reflected in the views on child sexuality, treated more as an asexual area and taboo. It is good to introduce the subject into public awareness because it will serve the practice well. Children will develop better, and in this way, an adult will be happy to realize their sexuality, and we clinicians might have less work.

Prof. Eleonora Bielawska-Batorowicz

On the subject of professional achievements, it can be assumed that publications, articles, or books are the results of our scientific work in psychology. I have quite a number of these publications to my credit and some books, but there are also some other exciting things we have been able to do. I say WE specifically because this is a shared achievement with my colleagues. On the one hand, I mean studies on prenatal bonds that, we could say, function to some extent as classical research in this area. Another interesting issue was the psychological examination of postpartum depression in men. I recall how my psychiatrist colleagues in the scientific institute told me that if I find one man with postpartum depression, they will describe me in the Express Ilustrowany (a regional newspaper that appears every day). With my then master’s female student, we found even more than one such person. We conducted the first Polish studies on postpartum depression in men, which are often quoted, and there are many references to our research in literature. They have even been included in the scientific review, so they are essential in this area.

I also conducted a psychological study on menopause, filling a gap in this area. My psychological monograph – aspects of menopause – offers knowledge to scientists and other people interested in the subject. However, what else seems interesting is that I used the Edinburgh Postnatal Depression Scale and created its Polish version, which was officially published as a Polish adaptation of the tool. It has started to be used in recommendations for doctors and midwives to study women in the perinatal period.

One of my most significant achievements is that theoretical knowledge has come into practice and is circulating among those practicing psychology as a practical discipline. It is also wonderful for the creator to see the effects of their work, which live in a world of science and impact real-life and help another person.

In turn, my most important scientific achievement is that I have stayed in science, despite various obstacles and difficult circumstances. It also seems that it is a great success that my research is objective and free from ideological and political entanglements. Also, I consider my stubbornness to do what I like and what gives me much satisfaction to be my outstanding achievement.

### What helped study participants in the development of their scientific career

All study participants highlighted the importance of mentoring and, in particular, female mentors for young women in science. According to the study participants, a young women scientist should have a female mentor who has already achieved success in the scientific and administration world. The mentor can provide support with her specialist knowledge and help the young scientist develop essential skills, such as leadership, self-confidence, transparent and non-verbal communication that shows high self-esteem, ability to enter the male-dominated environments, to respond to discriminatory behavior or unwanted attention, to seek support, and persevere in pursuit of a goal. The statements of the study participants that illustrate the issue described are presented below.

Prof. Bernadetta Izydorczyk

I was most helped by people from a close working environment in my career. I was very fortunate to meet people who helped me deal with difficulties in working as a psychologist and were happy to share their knowledge. For example, at the beginning of the clinical path in the psychologist profession, the heads of wards where I worked were men. I did not feel any discomfort for this reason. On the contrary, I even felt they encouraged and motivated me to work, although they also used my knowledge. This gave me a sense of security, and I knew I could count on my superiors. Then, at another workplace, I had a female head and female doctor colleagues who helped me very much. So I recall that period very well, too. Of course, I was aware of rivalry because it is always present, but I received much support from my colleagues at work nevertheless. I never felt that they had put skids under me.

In contrast, during my scientific work at the University of Silesia, I was very much supported mainly by female colleagues with higher scientific status. It was a kind of female mentoring I experienced at the time. I really have excellent memories of this cooperation, perhaps because there is no equal proportion between women and men in some environments. When I started as a director, I also received tremendous support from the academic community. I have been fortunate with staff, which positively impacted my career development.

At the same time, the relationship and understanding of my loved ones were extremely helpful. Sometimes they had to turn a blind eye to my absence when I was sitting at books or writing my doctorate. I also received much help from my husband. When I was focused on work, he had to mother our children. The family’s tremendous support and understanding of both my husband and daughters was very important.

Prof. Marta Bogdanowicz

My career began in difficult times due to the socialist political system. The problem of dyslexia in Poland was practically unknown then. During my first working period, I received little help from the academic community. Unfortunately, I did not have any support with my domestic responsibilities either. As far as helping to care for children was concerned, I had to deal with it myself. That is why my inner motivation was so important to me. The mission and the very willingness to create valuable solutions. This gave me much strength in difficult times, especially when writing my doctorate. In addition, both my mother and grandmother were incredibly energetic and energetic people. I also think that they were my role models as far as continuous activity and effective performance are concerned. To some extent, being a scout also helped me because I had to organize myself and others from the very beginning. My coordination skills would prove necessary in the future.

Although the Polish scientific community was quite harsh for me during my first work, I got help from other countries. I was supported by two scientists - foreigners - and this helped me stretch my wings and operate at an international level. Furthermore, this allowed me to enter an international scientific group, where, over 25 years, I could attend seminars in various countries and draw on information that was not available in Poland. Thanks to this experience, I founded the Polish Dyslexia Association, and this year, we celebrate the 30th anniversary, all of which is based on volunteering!

Prof. Stanisława Steuden

Many things have helped and continue to help me grow. I think that a certain amount of intellectual humility is essential. Science is so broad that it is impossible to know everything, even in my field. I feel that I do not know much about many issues yet, despite my age. Humility is essential because it allows people to go further.

Indeed, many of the people I met on my way have significantly impacted my research career. I cannot list them all, but I have to pay tribute to my late Maestra Professor Zenomena Płużek, who established the clinical psychology department at the Catholic University of Lublin. I was a student and prof. Płużek noticed and appreciated my clinical ambition. She became my mentor, and I owe her invaluable knowledge and support in my development. In general, many people from my environment played a significant role, which helped me to expand my interests. There was a time when I was interested in late adulthood in human development. This was due to the aging of my parents and my patients. In 1985, I became a member of the Scientific Committee of the University of the Third Age, and this is where I discovered the huge wealth of this phase of life.

Also, interacting with people who selected to have seminars with me played a significant role in developing my knowledge. Because I do not impose the scope of scientific work, I felt that if someone writes their thesis with me, I should at least partially explore the area concerned. For example, I cannot head a doctorate in a field I do not fully know. That is why my science students played an essential role in my career path.

In a way, thanks to my science colleagues, I owe my professorial title because they saw the professor in me more than I saw it myself. I do not feel the need to chase a scientific career, and because I did not care so much about academic titles, I did not take it too seriously. I did not feel that rush. That is why I did not stress about it. I simply submitted my papers and thought that if it all turned out ok, then great, but if not, then let it be. Maybe it is related to my religion. It was vital for me, as a believer, to entrust my life to God, that whatever God provides, I would agree with it. So I need to thank God for what I have and believe that He will give me strength with the new challenges.

Interestingly, study participants also mentioned their concerns about the support received from their families or its lack. This aspect related to both sharing domestic responsibilities and supporting their career development.

### Barriers for women in the field of psychology

All study participants were aware of the restrictions for women in the field of psychology. The barriers can be divided into three categories: visible and easy to see; subtle and indirect, operating from a latent level, demonstrated in the form of the only right view of the world or attitudes toward female scientists. The third category of barriers concerned the behavior of female scientists and their psychological qualities.

In the first category of barriers, the most frequently emerging aspect was the lack of support during parenthood, which afflicts more women than men in most countries. In addition, the scientists’ evaluation system does not consider the years when women did not publish or carry out research due to looking after children. Of course, women are not assessed during maternity or parental leave, but in the overall summary of their achievements (which is relevant, for example, when applying for grants), the years devoted to bringing up children are easily noticed. Moreover, the current pandemic will affect women more due to traditional social and family roles. Examples of the additional burden on women and their reasons are described below.

The second category of barriers covers the way women are treated in science, particularly by the male-dominated scientific community, and the need for women to adapt to the way they see the world and the man-created approach to science is considered the only right one. The following statements of the study participants provide a more accurate outlook on both categories of barriers.

The third category of barriers includes the behavior and characteristics of women in science, which prevent them from being promoted and holding leadership positions. The statements of the study participants that illustrate the issue discussed are presented below.

Prof. Márta Fülöp

The first thing is motherhood. I will give an example. When I got back to work (Hungarian Academy of Sciences) after 8 years of being at home with my three children, I was sitting with my young colleague who was a student with me at the University. He also got a job at the Academy of Sciences. I was always a better student than him. We were sitting close to each other and writing reports about our achievements in the previous year: how many articles we published, how many conferences we attended, etc. I just came back to the Academy, and he was all the time there, doing the research. He started to fill up the report. He was writing, filling up all of the rubrics, and I was sitting in front of my report, and most of the questions I had to leave unanswered because there was nothing to say. I literally almost started to cry because I felt that I was a much better student than him. I was at least as smart as him, and I had nothing to write. I always remember this. Now I have so many things to put into each rubric. I started later, but I caught up. Then I was much better than him.

The current pandemic situation affects women more than men. In many families, men do not want to put women in a subordinate role. Still, in reality, men earn more, so if you have to choose who can stay at home and be with the child, it will be a woman. Also, workplaces are more tolerant for women if they do not do their job 100% in the COVID situation. They are not that tolerant toward men. They would not accept when the man said: “I am sorry, I cannot work 100% in the home office because I have three kids and I have to school them.” They are going to hear: “Why does not your wife school them?” Also, generally, women do much more housework even if they are highly educated and work. If everybody is at home – you have to cook for them. Who is going to cook? Not a man in the home office but a woman. For those with small children, especially for women, COVID is a tough time, and it will affect women in academia more than men.

I notice that in committees where there are more men (but also when there are more women), there is extra attention and significance given to a remark or a suggestion if it comes from a man or a woman. This is something that maybe people are not even aware of. It just happens. They repeat something more often, remember it better, or think about it more if it comes from a man. If you want to be heard as a woman, you have to assert more, but if you assert more, you will be perceived as a cold and hardcore woman, while men are perceived as just confident if they behave the same way. Generally speaking, I can establish respect in such committees, and I am a member of many influential professional committees in Hungary, all maledominated (I mean there are more men than women). This is, in fact, an exciting combination. I can sometimes present my agenda better than a man, exactly because I am a woman. If I say something rational, a better suggestion than a man, he is more willing to accept it and take it as collaborative thinking, while from a man, it would be considered a rivalry situation. I learned how to avoid men starting to consider me as a rival instead of a female “partner.” If a woman can break the ignorance barrier, she has more chance than another man, as she is not considered a male rival.

I always had subtle difficulties with male doctoral students. They take me much less seriously than young male Ph.D. students take male supervisors. They grant much more respect to a male supervisor (even if they seem to be buddies) than to a female. It may be like a female boss for a male.

Tina Lindhard, Ph.D.

Women certainly have a more demanding role in academia than men. I must say, though, that has not been my case. or at least I do not think so. Any problems publishing my theories of consciousness I have always put down to differences in models (for example, neuroscientific view versus the idea of the deeper self and the heart) rather than my sex but maybe on second thoughts, there could be some overlap on a subtle level. However, I do feel men have convinced men to have a consensus view of reality rather than considering we might all view the grand mystery in unique and different ways.

Prof. Antonia Bifulco

I think that the disadvantage women have is firstly not seeing themselves as leaders or having the confidence to push. Women do not always have the confidence to apply for leadership positions and see themselves as leaders. Secondly, they stay at their jobs longer, and most people get a promotion by changing jobs. Of course, having children and maternity leave cause a break. Women tend to make statements that sound less confident, not to risk seeing themselves in charge of things, to differ from men in some situations. I think it has changed thanks to the educational system.

We found that girls always have lower self-esteem than boys when we studied self-esteem. But, on the other hand, boys have such high self-esteem that we could hardly place it on the scale we have got. So we asked: “What would you rate yourself?” They said: “100%.” And you think: “Are they joking?,” but no, they thought they were perfect.

Prof. Maria Beisert

Ostracism can be a barrier. However, I am not very interested in the ostracism of specific institutions. For example, I am completely not interested in the approval of what I write about human sexuality by the Catholic Church. And I quite calmly tolerate the rejection of this institution. I do not think this is courage; I see it as my independence. I want to give people the wealth and pleasure of having deep, intimate relationships with another person and to enjoy those relationships.

Prof. Lidia Cierpiałkowska

Regarding the barriers to women’s scientific development in psychology, I am reminded of the times when I started learning. Psychology was one of the most demanding courses of study. I heard at that time that I should not dream of it at all because it was impossible to get into psychology. There were many applicants, 17–20 people per one place, but I managed to get it and started learning in the dream field. One hundred twenty people were studying with me, roughly equally men and women. Nevertheless, when the professors entered the class, they said:

“So many women here. You should have stayed at home, marry well. Cook and care for your children.” This, of course, only increased our (women’s) determination to continue studying and emancipating at that time. When I think about this question, I also think of unpleasant situations. During these times, various professors invited students to their cabinets, and various things went on there. I do not know this from my own experience, but I heard a lot from my female colleagues. As a female student at that time, it was difficult to oppose the professor. It seems to me that these things do not happen at present too often, but rather somewhat by accident. I personally did not experience any of this. I would say that I was treated the way I let others treat me. A relationship with a man at university, at work or in other situations is a social relationship where one should know what she wants and what she expects from such a relationship. It is essential to define the limits and consistently enforce them.

On the other hand, referring to the current barriers, I note the negative consequences of the COVID-19 pandemic. They are related to the overload of women, who, on the one hand, care for the home child education and, on the other, are also involved in remote learning and are trying to carry out some scientific work. Unfortunately, they often have the time for that after all other family members go to bed. I think that this is a considerable challenge for a woman, especially one that works in a traditional family model, where the husband is helping to a limited extent with domestic chores because his work and career are more important than the wife’s work. It also happens that the help of grandparents in caring for children must be limited because of the fear of infection. Therefore, it seems to me that some women are making a sort of revision of their lives, about what is important to them and what they want to spend their time on, just as is the case in emergencies. In other words, they are faced with a particular choice, and I believe that this will primarily be developmental choices.

It is worth noting that the study participants’ observations on the limitations and barriers concern either their entire working life, therefore signifying their variety, or current limitations due to their psychology area of expertise or the situation arising from, for example, COVID.

### Guidelines and recommended actions to promote equal opportunities for women in science

The study participants indicated two types of guidelines and recommended actions that promote equal opportunities for women in science. The first type refers to developing the structures that can permanently support women in science through transformative, deep occupational programs that help diagnose their key talents, needs, and desires. The second is systemic solutions to be implemented by universities and legal solutions that could align women’s opportunities in science, increase their access to research funding, and the success of publications. The statements of the study participants that illustrate the issue discussed are presented below.

Prof. Gopa Bhardwaj

The advice I would give to women deciding to pursue a career in science would be: be sincere. Do not do the job for the sake of doing the job. Be committed. Have some love for your understanding and knowledge. Extend that love on your recipients, students, colleges, and people close to you. And have patience because being a woman in science, especially in administration, is not very easy. If you want to work in an administrative position, make sure you are a little dominating. Tell your views, put your foot down. That is only possible when you are sure of your standing. So that is the commitment, the sincerity, the love for the job. If you have all these, then nobody can stop you. It may be difficult, but it is possible. Believe what you are doing and have this confidence to tell that this is what I am doing and why I am doing it, and this is how I am going to do it. Be transparent. Unfortunately, women often do not have enough self-confidence to do it. They are part of the flock. They need to be guided. They are not very self-confident, do not value their achievements, and do it mechanically. Do your job with heart. Easier said than done but possible. Bring science close to life and work for the welfare of humankind. Science and values have to go together, the existential values. All scientific research is useless if they do not carry meaning.

Prof. Eleonora Bielawska-Batorowicz

Regarding the issue of equal opportunities for women in science, I would like to point out that our attitudes often cause many gender inequalities. Therefore, if I were to recommend anything to women in science, I would say that, above all, they need to know and never doubt that they are as good as men. They should not say that they cannot do something in their professional career because of their gender! This is my advice: women should remember that they can do everything they can, or even better. They should not allow being pushed out of the academic circles to the roles of the service.

In addition, I would like to see women being aware of and prepared to have to choose sometimes. These can be tough choices at times. It is often more difficult for them than for men because they have to decide when to set up a family and reconcile work and private life.

It should also be emphasized how important it is to allow yourself time to grow. Take advantage of all the opportunities offered by modern science: internships, scholarships, publications, international conferences, and other things to do for our own development. But, of course, this is not a simple matter because it is also a question of finances, which needs to be taken care of early on.

It is worth noting that scientific work is, in a sense, a race that does not have a finish line and never ends. I have a cup at home with a rat race drawing, and one of these wise rats says to others: “Remember, my dear ones, there is no finishing line.” Therefore, an important aspect is also a particular approach to learning how to play, in the sense that the work we do gives us joy and satisfaction so that we can enjoy it every day.

Prof. Marta Bogdanowicz

The advice I could give young women starting a research job is that if they are fascinated by the subject, they should go in that direction. Cognitive curiosity is always a source of progress and the particular joy of work. You should not give up your goals when something goes wrong because you will succeed tomorrow if not today! I believe that you feel the most excellent satisfaction and fulfillment when science can be used for practical purposes and benefits other people. The usefulness of our work is what gives you wings, tremendous energy and is the best motivation to continue to work.

I believe that there could also be some solutions at the institute level to support women in scientific development. However, most of us women need to go through the period of family building, children’s joy, and here lies vital support. At present, the institution of “grandmother” has already been exhausted. The model from the past years that we used to count on - the grandmother taking care of the children - is, unfortunately, no longer applicable. Now, grandmothers attend the universities of the third age. They are often active, engaged in sports, and enjoy their own life. Therefore, it is essential, first and foremost, that good institutions such as professional nurseries and preschools are available, which can ease the burden on us throughout the day. In our country, nurseries often seem to be a terrible place, which makes us think that, until they are three, our children should only be at home. This is not true if there is a good nursery with wise, competent, and friendly carers who work well with children and their parents. This could be a great space for the development of the little ones. As women, we must feel a sense of security that we leave children in good hands. It is challenging to work effectively without the comfort that our children are properly cared for and have the best educational conditions.

## Discussion and conclusions

Our interviews with women scientists presented similar narrative stories about experiencing specific difficulties in developing their scientific and administrative careers at universities. Therefore, we feel that the information provided by our interlocutors can be the answer to why the number of women is decreasing as they progress through scientific development (Women in Science Report^1^ or Women in Science, 2020).

The common thread in the statements of women scientists was the combination of professional and family responsibilities, which results from environmental, religious, or cultural requirements. It can be said that the combination of a professional and family path is, on the one hand, a “delaying” factor in the performance of professional tasks, but, on the other hand, a “buffer” that allows finding oneself in the surrounding reality. Their history is characterized by the expression of purpose and meaning, which is identity-formative. The statements of our interlocutors are supported, for example, by the findings of the European Commission’s report “She Figures” – it is generally more difficult for women than men to step up the career ladder.

In addition, some women attribute their achievements to hard work and the “struggle” for recognition because their activities are very often inscribed in culturally sanctioned “women’s” attitudes (care, feelings, emotions), social roles (assistants, lecturers), or problem areas (children, family), (Derra et al., 2021).

In most cases, the study participants referred to the non-egocentric motivation that stems from values such as universalism and benevolence: the desire to bring valuable and helpful things to the lives of others, to share something that they discovered and considered to be necessary, courageously promote unpopular knowledge, despite the risk of being intimidated in the scientific world, and a desire to care for their families. This attitude shows the study participant’s extraordinary social and moral maturity but may also result from the construction of what is female and what is male. These social and cultural constructions of male and female attributes and roles may cement the spaces that women can have on the career ladder (Febbraro, 2020; Rutherford, 2020; Skalski and Pochwatko, 2020).

Moreover, the study participants strongly emphasized the practical aspect of their research. The development of theory, psychological concepts, or paradigms was not so important to them, as was applying the research in practice (the usefulness of what they do for others). However, power or influence over other persons has no significance for them. It may be due to the barrier identified by our interlocutors regarding how women are treated in science, particularly by the male-dominated scientific community, and the need for women to adapt to the way they see the world and the man-created approach to science (Chybicka and Zubrzycka, 2015; Kirschner, 2020; Derra et al., 2021). Perhaps that is why our interlocutors highlighted mentoring offered by female mentors and its importance to young women in science. They stressed the need for support in terms of expertise and skills such as leadership, self-confidence, ability to enter male-dominated environments, respond to discriminatory behavior or comments, seek support, and persevere in pursuit of their goals. On this occasion, it is essential to ask how growing these characteristics and behaviors results from the personal experience of our interlocutors, how specific it is for the women psychological scientists, and to what extent it results from changes in women’s awareness due to socio-cultural changes? It is also difficult not to refer to the observation made by Ceci and Williams (2011), who pointed out that adolescent girls often prefer a career focused on people, which in turn translates into a growing number of women in areas such as medicine and biology (it should also be assumed that the same applies to psychology). The authors mentioned above stress the role of cultural patterns in science imposed not only on femininity but also the potential for scientific/professional development of women. Rutherford (2020) also indicates the importance of the historical aspect of the “hard” sciences like mathematics with masculinity, and “soft” sciences, like biology and psychology with femininity.

Following our study, we come to the conclusions reflected in the literature on the subject (Ceci and Williams, 2011; Kirschner, 2020; Osbeck, 2020; Derra et al., 2021), namely, that the promotion of women in psychology or, more generally, in science can take place at least two ways. First, through the conscious undertaking of individual actions, we value female researchers, express our admiration for their scientific activities, and restore them to the history and memory of the various research fields. Secondly, through system-based solutions that aim to transform science mechanisms into more women-friendly ones (e.g., taking into account periods of absence from childcare).

When drawing study conclusions, it is worth noting all of study participants were Europeans but one participant was an Indian from Asia. The authors did not observe any specificity in their statements compared to the other participants. The categories of experience described seem to be supra-cultural. In contrast, based on the participants’ statements, the authors observed that the more eastward the country of origin of the study participants, the less support from partners (men) the study participants received and the greater the need to combine the stereotypical female roles with the work. This was especially true for women who had children. There may be a possible relationship between the level of masculinity and collectivism of the culture and religious and cultural differences. However, reliable conclusions are difficult due to the over-representation of Polish women scientists in the study. In future, the study should be extended to persons from other cultural circles and cover women scientists from all possible continents.

Guidelines for equality programs form an important element of the study. The role of women mentors in such programs is particularly interesting in the authors’ opinion. Study participants stressed that it was important to learn from other women who were open and willing to support the mentees. Many study participants mentioned being a victim of the reluctance of other researchers. This was more often the case for male mentors, but it resulted from the fact that there were not many women mentors in science when the study participants were mentees. However, many women also experienced support from male mentors, and all have stressed the role of an experienced mentor and the importance of women’s mentorship. Women mentors act as role models and show that success for women at high-level jobs is possible.

Another essential premise for equality programs is that they not only contain knowledge elements but that, through practical activities, workshops, and psychological work themselves, they transform participants at a deeper level, particularly by affecting their self-assessment. According to the interviewees, women often lack self-confidence, and high, stable self-assessment is a strong predictor of the success of women in science.

## Data availability statement

The raw data supporting the conclusions of this article will be made available by the authors, without undue reservation.

## Ethics statement

Ethical review and approval was not required for the study on human participants in accordance with the local legislation and institutional requirements. The patients/participants provided their written informed consent to participate in this study. Written informed consent was obtained from the individual(s) for the publication of any potentially identifiable images or data included in this article.

## Author contributions

BP-W: study design, data collection, data interpretation, manuscript preparation, and literature search. AC: data collection, data interpretation, manuscript preparation, and literature search. Both authors contributed to the article and approved the submitted version.

## Footnotes

1
http://uis.unesco.org/en/topic/women-science

## References

[B1] Ceci S. J. Williams W. M. (2011). Understanding current causes of women’s underrepresentation in science. *Proc. Natl. Acad. Sci. U.S.A.* 108 3157–3162. 10.1073/pnas.1014871108 21300892 PMC3044353

[B2] Ceci S. J. Williams W. M. Barnett S. M. (2009). Women’s underrepresentation in science: Sociocultural and biological considerations. *Psychol. Bull.* 135 218–261. 10.1037/a0014412 19254079

[B3] Chase S. E.. (ed.) (2009). *Wywiad narracyjny. Wielośæ perspektyw, podejśæ, głosów.* Warszawa: Wydawnictwo Naukowe PWN.

[B4] Chybicka A. Zubrzycka E. (2015). *Siła kobiet w biznesie. Czego możesz się nauczyć od 10 kobiet biznesu.* Gdańsk: Gdańskie Wydawnictwo Psychologiczne.

[B5] Ćwiklicki M. Urbaniak A. (2018). Methodological rigour in descriptions of case study research in polish academic articles on management. *Przedsiębiorczość Zarządzanie* 6 165–177.

[B6] Derra A. Kola A. Piasek W. (2021). *Niewidzia(l)ne. Kobiety i historia uniwersytetu mikołaja kopernika w toruniu.* Toruń: Wydawnictwo Naukowe Uniwersytetu Mikołaja Kopernika.

[B7] Febbraro A. R. (2020). Critical feminist history of psychology versus sociology of scientific knowledge: Contrasting views of women scientists? *J. Theo. Philos. Psychol.* 40 7–20. 10.1037/teo0000133

[B8] Hałas E. (1990). “Biografia a orientacja symbolicznego interakcjonizmu,” in *Metoda biograficzna w socjologii*, eds Włodarek J. Ziółkowski M. (Warszawa: Wydawnictwo Naukowe PWN), 197–208.

[B9] Hänninen V. (2004). A model of narrative circulation. *Narrat. Inq*. 14 69–85. 10.1075/ni.14.1.04han 33486653

[B10] Hoerning E. (1991). *Biographieforschung und erwachsenenbildung.* Bad Heilbrunn: Verlag Julius Klinkhardt.

[B11] Hurrelmann K. (1994). *Struktura społeczna a rozwój osobowości.* Poznań: Wydawnictwo Naukowe UAM.

[B12] Kirschner S. R. (2020). Commentary. What does it mean to say science is gendered? *J. Theo. Philos. Psychol.* 40 54–57. 10.1037/teo0000136

[B13] Osbeck L. M. (2020). Lost and found in the margins: Women, interdisciplinary collaboration, and integrative development. *J. Theo. Philos. Psychol.* 40 32–42. 10.1037/teo0000132

[B14] Piorunek M. (1996). *Dziecko w relacjach ze światem zawodowym.* Poznań: Eruditus.

[B15] Rutherford A. (2020). Doing science, doing gender: Using history in the present. *J. Theo. Philos. Psychol.* 40 21–31. 10.1037/teo0000134

[B16] Skalski S. Pochwatko G. (2020). Gratitude is female. Biological sex, socio-cultural gender versus gratitude and positive orientation. *Curr. Issues Pers. Psychol.* 8 1–9. 10.5114/cipp.2020.93624

[B17] Stemplewska-Żakowicz K. (2002). “Koncepcje narracyjnej tożsamości,” in *Narracja jako sposób rozumienia świata*, ed. Trzebiński J. (Warszawa: Warszawskie Wydawnictwo Literackie MUZA), 81–113.

[B18] Stemplewska-Zakowicz K. (2010). Metodyjakosciowe, metodyilosciowe: Hamletowskidylematczyróznorodnosc do wyboru?. *Ann. Psychol.* 13, 87–96.

[B19] Stemplewska-Zakowicz K. Krejtz K. (2005). *Wywiadpsychologiczny: Wywiadjakopostepowaniebadawcze*. Warszawa: PracowniaTestówPsychologicznychPolskiegoTowarzystwaPsychologicznego.

[B20] Tyszkowa M. (1988). *Rozwój psychiczny człowieka w ciągu życia.* Warszawa: Wydawnictwo Naukowe PWN.

[B21] Wengraf T. (2012). “Interpretacja historii Życiowych, sytuacji Życiowych i doświadczeń osobistych: Biograficzno-narracyjna metoda interpretacyjna (BNIM-Biographical-Narrative Interpretative Method),” in *Metoda biograficzna w socjologii. Antologia tekstów*, ed. Kaźmierska K. (Kraków: Nomos), 351–362.

[B22] Włodarek J. Ziółkowski M. (eds). (1990). *Metoda biograficzna w socjologii.* Warszawa: PWN.

[B23] Women in Science (2020). *Diversity management and gender equality in social responsibility of University of Gdańsk.* Available Online at: https://ug.edu.pl/news/en/421/ug-report-women-science-managing-diversity-and-gender-equality-within-social-responsibility [accessed September 22, 2020].

[B24] Wyer M. (2018). “In the company of feminist science,” in *APA handbook of the psychology of women: Perspectives on women’s private and public lives*, eds Travis C. B. White J. W. Rutherford A. Williams W. S. Cook S. L. Wyche K. F. (Washington, DC: American Psychological Association), 459–474. 10.1037/0000060-025

